# Inactivation of ERK1/2 signaling mediates dysfunction of basal meningeal lymphatic vessels in experimental subdural hematoma

**DOI:** 10.7150/thno.87633

**Published:** 2024-01-01

**Authors:** Jiangyuan Yuan, Xuanhui Liu, Meng Nie, Yupeng Chen, Mingqi Liu, Jinhao Huang, Weiwei Jiang, Chuang Gao, Wei Quan, Zhitao Gong, Tangtang Xiang, Xinjie Zhang, Zhuang Sha, Chenrui Wu, Dong Wang, Shenghui Li, Jianning Zhang, Rongcai Jiang

**Affiliations:** 1 Department of Neurosurgery, Tianjin Medical University General Hospital, Tianjin 300052, China.; 2 Tianjin Neurological Institute, Key Laboratory of Post Neuro-injury Neuro-repair and Regeneration in Central Nervous System, Ministry of Education and Tianjin, Tianjin 300052, China.; 3 Department of Rehabilitation Medicine, The Second Affiliated Hospital of Anhui Medical University, Hefei 230601, China.; 4 State Key Laboratory of Experimental Hematology, Tianjin Medical University General Hospital, Tianjin 300052, China.

**Keywords:** subdural hematoma, meningeal lymphatic vessels, meningeal lymphatic drainage, ERK1/2, atorvastatin

## Abstract

**Rationale**: Meningeal lymphatic vessels (MLVs) are essential for the clearance of subdural hematoma (SDH). However, SDH impairs their drainage function, and the pathogenesis remains unclear. Herein, we aimed to understand the pathological mechanisms of MLV dysfunction following SDH and to test whether atorvastatin, an effective drug for SDH clearance, improves meningeal lymphatic drainage (MLD).

**Methods**: We induced SDH models in rats by injecting autologous blood into the subdural space and evaluated MLD using Gadopentetate D, Evans blue, and CFSE-labeled erythrocytes. Whole-mount immunofluorescence and transmission electron microscopy were utilized to detect the morphology of MLVs. Phosphoproteomics, western blot, flow cytometry, and *in vitro* experiments were performed to investigate the molecular mechanisms underlying dysfunctional MLVs.

**Results**: The basal MLVs were detected to have abundant valves and play an important role in draining subdural substances. Following SDH, these basal MLVs exhibited disrupted endothelial junctions and dilated lumen, leading to impaired MLD. Subsequent proteomics analysis of the meninges detected numerous dephosphorylated proteins, primarily enriched in the adherens junction, including significant dephosphorylation of ERK1/2 within the meningeal lymphatic endothelial cells (LECs). Subdural injection of the ERK1/2 kinase inhibitor PD98059 resulted in dilated basal MLVs and impaired MLD, resembling the dysfunctional MLVs observed in SDH. Moreover, inhibiting ERK1/2 signaling severely disrupted intercellular junctions between cultured LECs. Finally, atorvastatin was revealed to protect the structure of basal MLVs and accelerate MLD following SDH. However, these beneficial effects of atorvastatin were abolished when combined with PD98059.

**Conclusion**: Our findings demonstrate that SDH induces ERK1/2 dephosphorylation in meningeal LECs, leading to disrupted basal MLVs and impaired MLD. Additionally, we reveal a beneficial effect of atorvastatin in improving MLD.

## Introduction

Subdural hematoma (SDH) is a prevalent form of intracranial hematoma, characterized by significant rates of disability and mortality 1, 2. It primarily arises from the rupture of bridging veins following cerebral trauma 3. The accumulation of hematoma within the subdural space adversely affects neural function, manifesting as intracranial hypertension and brain tissue displacement 4, 5. Prolonged hematoma accumulation can trigger inflammatory angiogenesis within the subdural space, leading to persistent blood leakage and chronic subdural hematoma (CSDH) development 6. Therefore, timely drainage of hematoma from the subdural space is crucial in SDH treatment. A proportion of clinical SDH cases can undergo spontaneous absorption 7, indicating the presence of a clearance pathway for SDH. Macrophages can phagocytize intracranial hematomas 8, but their infiltration also stimulates the formation of immature leaky blood vessels, contributing to the progression of CSDH 6. In contrast, the meningeal lymphatic system exhibits a powerful drainage function for intracranial hematomas and is clearly involved in SDH resolution 9-11.

The lymphatic system plays a crucial role in maintaining homeostasis in mammals by facilitating the elimination of physiological metabolites and pathological molecules 12. In recent years, neurological rediscoveries have revealed a previously neglected lymphatic vessel network within the dural mater transports intracranial metabolites to cervical lymph nodes (CLNs) 13, 14. Subsequent studies demonstrate that meningeal lymphatic vessels (MLVs) are implicated in the pathological progression of neurological diseases, especially in intracranial hemorrhage, traumatic brain injury (TBI), and Alzheimer's disease (AD) 9, 11, 15, 16. Targeting the lymphangiogenesis signaling pathway to strengthen meningeal lymphatic drainage (MLD) has shown improvements in these conditions. Conversely, weakening MLD by ligating the drainage route, or ablating MLVs, leads to worsening outcomes 9, 11, 15-17. These findings highlight the significant role of MLVs in cleaning neurotoxic substances like erythrocytes and suggest that targeting MLVs could be an effective intervention for neurological diseases.

Consistent with these studies, we previously revealed that hematoma accumulated in the subdural space could be drained into the deep cervical lymph nodes (dCLNs) via MLVs 10. Notably, ligating the lymphatic vessels upstream of the dCLNs resulted in significantly reduced hematoma absorption, indicating MLVs are vital routes for SDH clearance. In addition, we observed impaired MLD in the acute phase following SDH 10. Impaired MLD has been observed in multiple neurological disorders, such as TBI, subarachnoid hemorrhage (SAH), Parkinson's disease, and AD 15, 16, 18, 19. Although restoring the impaired MLD has shown a potential to enhance neurological outcomes, the underlying pathological alterations and molecular mechanisms of dysfunctional MLVs remain poorly understood, especially in SDH.

Understanding the pathological mechanisms of MLVs after SDH and repairing MLD are meaningful in SDH treatment. Atorvastatin, a classical lipid-lowering drug, has shown therapeutic effects in patients with CSDH in previous studies 20-22. *In vivo* experiments demonstrate that atorvastatin can alleviate subdural inflammation and promote the absorption of SDH 23, 24. In particular, atorvastatin can suppress lymphedema and preserve lymphatic drainage in the periphery 25. However, little is known whether the benefits of atorvastatin in SDH treatment are associated with MLD improvement. In this study utilizing SDH rat models, we aimed to investigate the pathological and molecular mechanisms underlying dysfunctional MLVs following SDH and examine the potential ameliorative effects of atorvastatin on MLD.

## Results

### Valved MLVs at the skull base drain subdural substances to dCLNs

**Figure 1:**
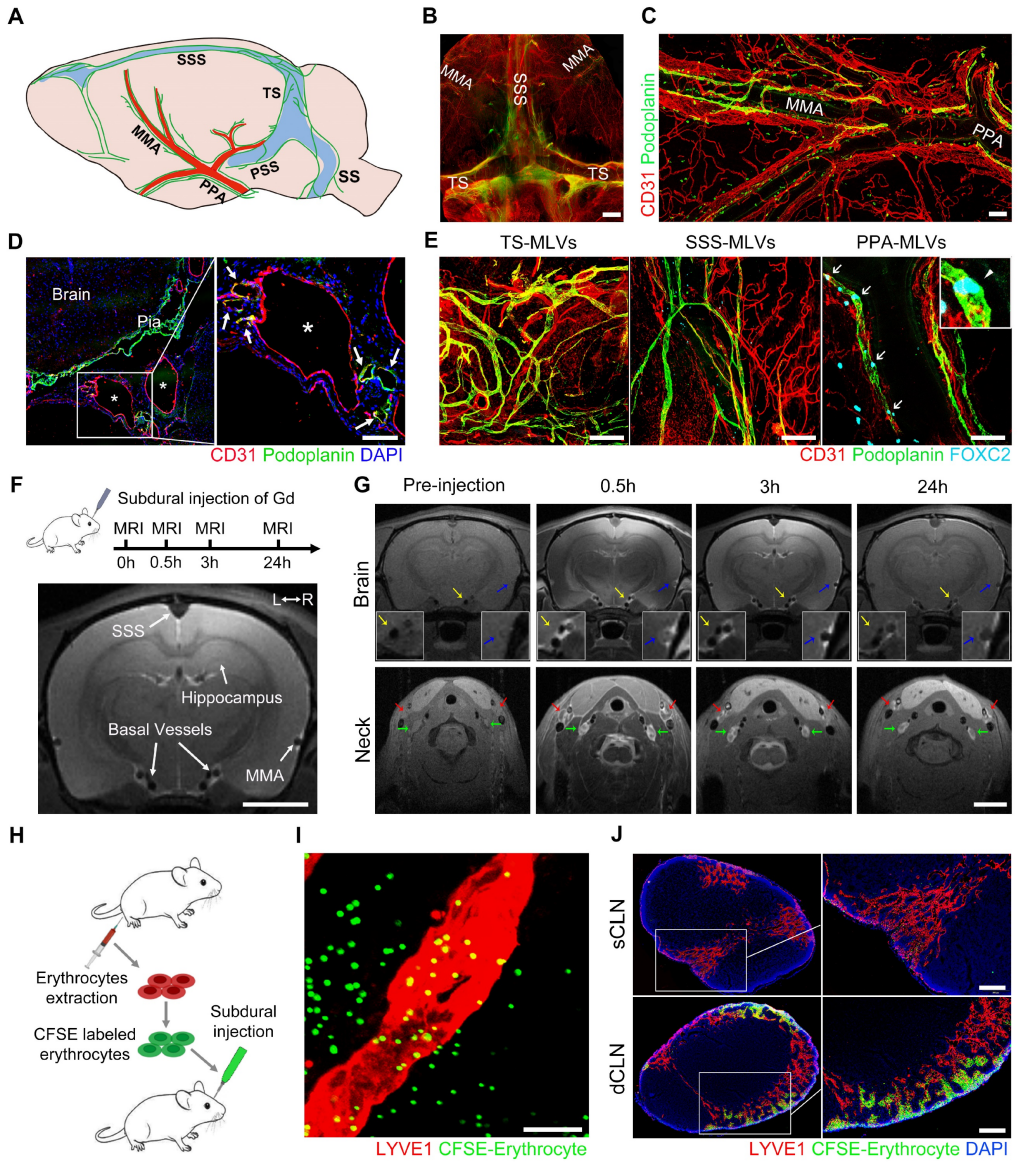
**Subdural substances drain to the CLNs through the basal valved MLVs. (A)** Schematic diagram of MLV system in rats. **(B)** Whole-mount immunostaining of the dorsal MLVs and** (C-D)** the basal MLVs surround the MMA, PPA, and basal vessels. White asterisks represent basal vessels; white arrows indicate MLVs. **(E)** Immunostaining of lymphatic valves within the MLVs with FOXC2 marker. White arrows indicate valves. **(F)** Scheme of Gd injection and diagram of the coronal brain. **(G)** Gd drainage process observed in the brain and CLNs. Blue arrows indicate MMA; yellow arrows indicate basal meningeal arteries; green arrows indicate dCLNs; red arrows indicate sCLNs. **(H)** Schematic diagram of subdural injection of CFSE labeled erythrocytes. **(I)** Basal MLVs adjacent to the PPA drain the subdurally injected erythrocytes into **(J)** the CLNs. Scale bars: **B** 2 mm; **C-E** 100 μm; **F-G** 1 cm; **I** 20 μm; **J** 200 μm. PSS: petrosquamosal sinus; SS: sigmoid sinus.

Prior to investigating the dysfunctional MLD following SDH, we ascertained the distribution of MLVs in rats and investigated the drainage characteristics of subdural substances. By employing the specific lymphatic marker podoplanin, we conducted immunostaining of the whole meninges and observed a complex lymphatic network in both the dorsal and basal meninges (Figure 1A). The podoplanin+ lymphatic vessels primarily distribute surrounding the sinuses and major arteries within the dura mater. Previous studies identify these lymphatic vessels surrounding the superior sagittal sinus (SSS) and transverse sinus (TS) as dorsal MLVs (Figure 1B) 26. In contrast, those running alongside the middle meningeal artery (MMA) and basal vessels are classified as basal MLVs (Figure 1C-D) 26, 27. Our investigation further revealed the absence of FOXC2+ lymphatic valves within the dorsal MLVs. In contrast, the basal MLVs adjacent to the pterygopalatine artery (PPA), a vital artery connecting the MMA to the external carotid artery, exhibited abundant valves (Figure 1E). Additionally, unlike the intricate lymphatic network exhibited in the dorsal meninges, the basal MLVs were orderly arranged alongside the arteries, devoid of excessive branching. These distinct structures of the basal MLVs are more efficient in facilitating unidirectional lymph transport 12, 28.

Subsequently, we injected Gadopentetate D (Gd) contrast agents into the subdural space of rats and employed 9.4 T magnetic resonance imaging (MRI) to detect their drainage pathways (Figure 1F). These injected Gd rapidly flowed into the skull base and enriched around the basal vessels, which were bilaterally accompanied by MLVs. Correspondingly, a large amount of Gd drained and accumulated in the downstream CLNs (Figure 1G). Although the Gd exhibited in all the CLNs, a more pronounced contrast signal was observed in the dCLNs. This finding was further corroborated by the subdural injection of Evans blue (EB), detecting a higher concentration of EB in the dCLNs (Figure S1A-B). In addition to the observed Gd drainage within the basal MLVs, we further observed the infiltration of Gd into the cavity surrounding the TS. Conversely, no contrast agents were detected within the cribriform plate (Figure S1C-E).

To verify whether erythrocytes, the main component of SDH, could be directly drained through the basal MLVs. We labeled erythrocytes with a fluorescent dye CFSE and injected them into the subdural space (Figure 1H). After 30-minute drainage, CFSE+ erythrocytes were detected in the LYVE1+ lymphatic lumen adjacent to the PPA (Figure 1I). In consist with Gd and EB, these CFSE+ erythrocytes primarily drained into the dCLNs (Figure 1J). The above findings revealed that subdural substances predominately drained into the dCLNs via the MLVs system, highlighting the role of valved basal MLVs as essential drainage routes.

### SDH induces impaired MLD and disrupted basal MLVs on the ipsilateral side

**Figure 2:**
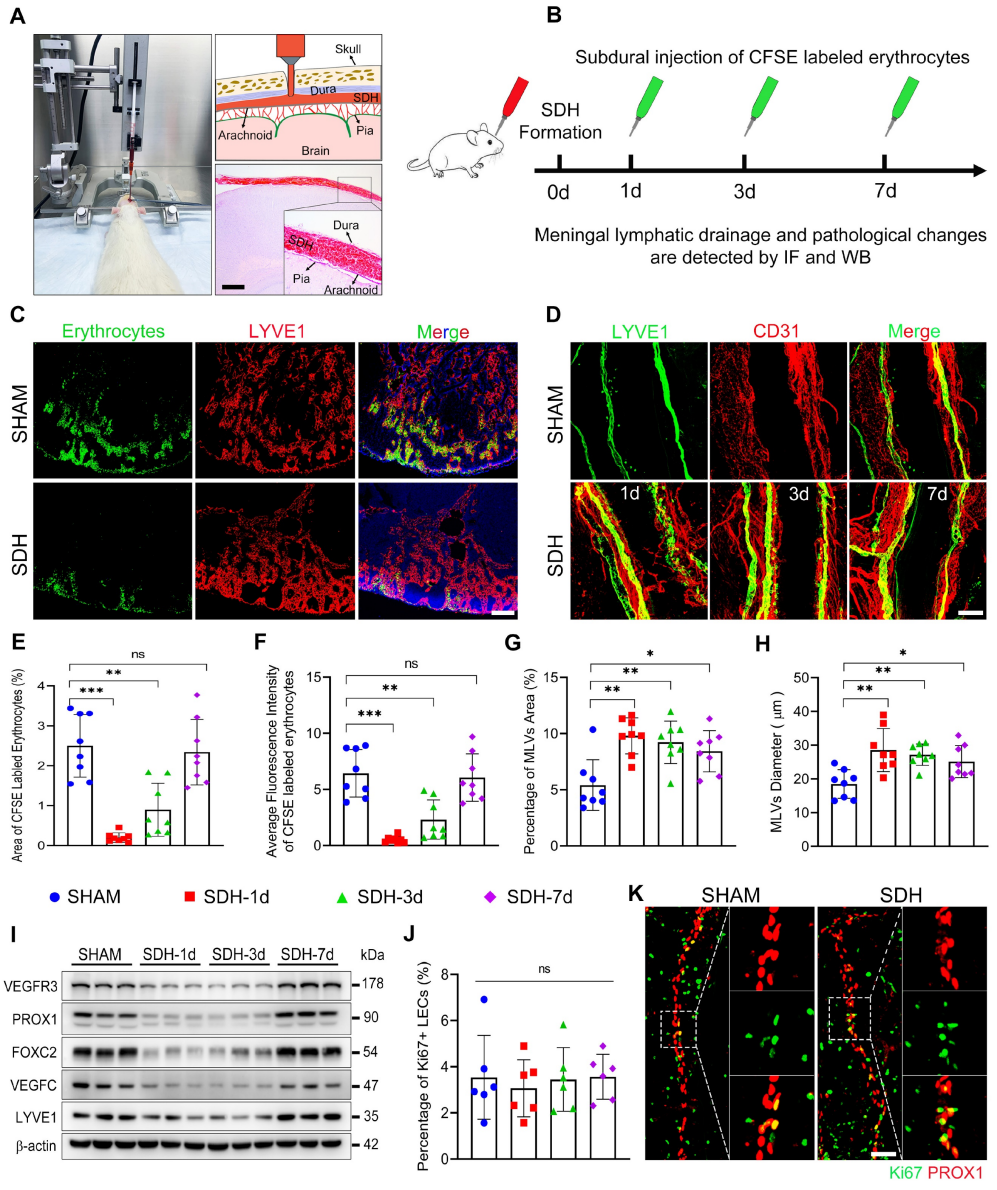
** SDH induces impaired MLD and pathological dilation in the basal MLVs. (A)** Schematic diagram of SDH formation and **(B)** experimental procedures. **(C)** Drainage efficiency of CFSE labeled erythrocytes detected in the ipsilateral dCLNs. The exhibited pictures are photographed 30 min after subdural injection on the third day following SDH. **(D)** Immunostaining of LYVE1+ basal MLVs adjacent to the ipsilateral PPA. The CFSE+ area percentage **(E)** and average fluorescence intensity of CFSE+ erythrocytes **(F)** in the ipsilateral dCLNs. n = 8 rats per group. Quantifying the coverage area **(G)** and lumen diameter **(H)** of LYVE1+ MLVs adjacent to the ipsilateral PPA. n = 8 rats per group. **(I)** Western blot analysis of lymphatic marker protein expressions in the meninges following SDH. n = 3 rats per group. **(J-K)** Assessing meningeal lymphangiogenesis via Ki67+ LEC staining. n = 6 rats per group. Data are presented as means ± SD, statistical analysis with one-way ANOVA followed by Dunnett post hoc test in **E-H, J**. * P < 0.05; ** P < 0.01; *** P < 0.001; ns, no significance. Scale bars: **A** 1 mm; **C** 200 μm; **D** 100 μm; **K** 50 μm.

Subsequently, we formed SDH in rats by injecting autologous blood into the subdural space (Figure 2A). On the first, third, and seventh-day following SDH, we re-injected CFSE-labeled erythrocytes or EB into the subdural space to assess the efficacy of MLD (Figure 2B, S2A). Following 30-minute drainage, all dCLNs were harvested. Compared to the sham group, the accumulation of EB or CFSE-labeled erythrocytes in the dCLNs reduced on the first and third days following SDH (Figure 2C,E-F, S2B-C). Remarkably, the decreased drainage of subdurally injected dye primarily manifested in the ipsilateral dCLNs, with no significant difference observed in the contralateral dCLNs (Figure S2D). The impaired MLD exhibited a gradual restoration over time and fully returned to normal drainage efficiency by the seventh day. Moreover, a higher content of EB drained into the ipsilateral dCLNs in the sham group, indicating an ipsilateral drainage tendency of MLD (Figure S2B,E-F). The impaired MLD has been demonstrated to correlate with increased intracranial pressure (ICP) 15, 29. However, we found that the MLD efficiency remained unaffected in rats subjected to subdural injection of equal-volume saline (Figure S2E-F). Despite an initial surge in ICP during the injection process, it gradually declined and stabilized at the pre-injection baseline within hours (Figure S2G-I). Therefore, the impaired MLD following SDH suggested underlying pathological alterations within the MLVs.

Next, we stained the whole meninges with lymphatic markers, but no noticeable change was detected on the dorsal MLVs. The LYVE1+ lymphatic area surrounding the TS was similar throughout the period of SDH absorption (Figure S3A-B). Conversely, the basal MLVs exhibited significant morphological alterations. Concurrent with the impaired MLD, the areas of MLVs surrounding the ipsilateral PPA increased obviously, accompanied by enlarged diameters of the lymphatic lumen (Figure 2D,G-H). Notably, these alterations were not detected in the contralateral basal MLVs (Figure S3A,C-D). To ascertain whether the expansion of MLVs was compensatory lymphangiogenesis to facilitate the absorption of SDH, we assessed the expression levels of various lymphatic marker proteins in the meninges. However, following SDH, there was no increase in the expressions of these proteins, including LYVE1, PROX1, FOXC2, VEGFC, and VEGFR3 (Figure 2I). Furthermore, the expression of FOXC2 and PROX1, two crucial structural proteins of lymphatic valves 26, 30, decreased in the acute phase following SDH. Ki67 staining, a specific marker for proliferation, further corroborated that the dilated lymphatic lumen was unrelated to lymphangiogenesis (Figure 2J-K).

**Figure 3:**
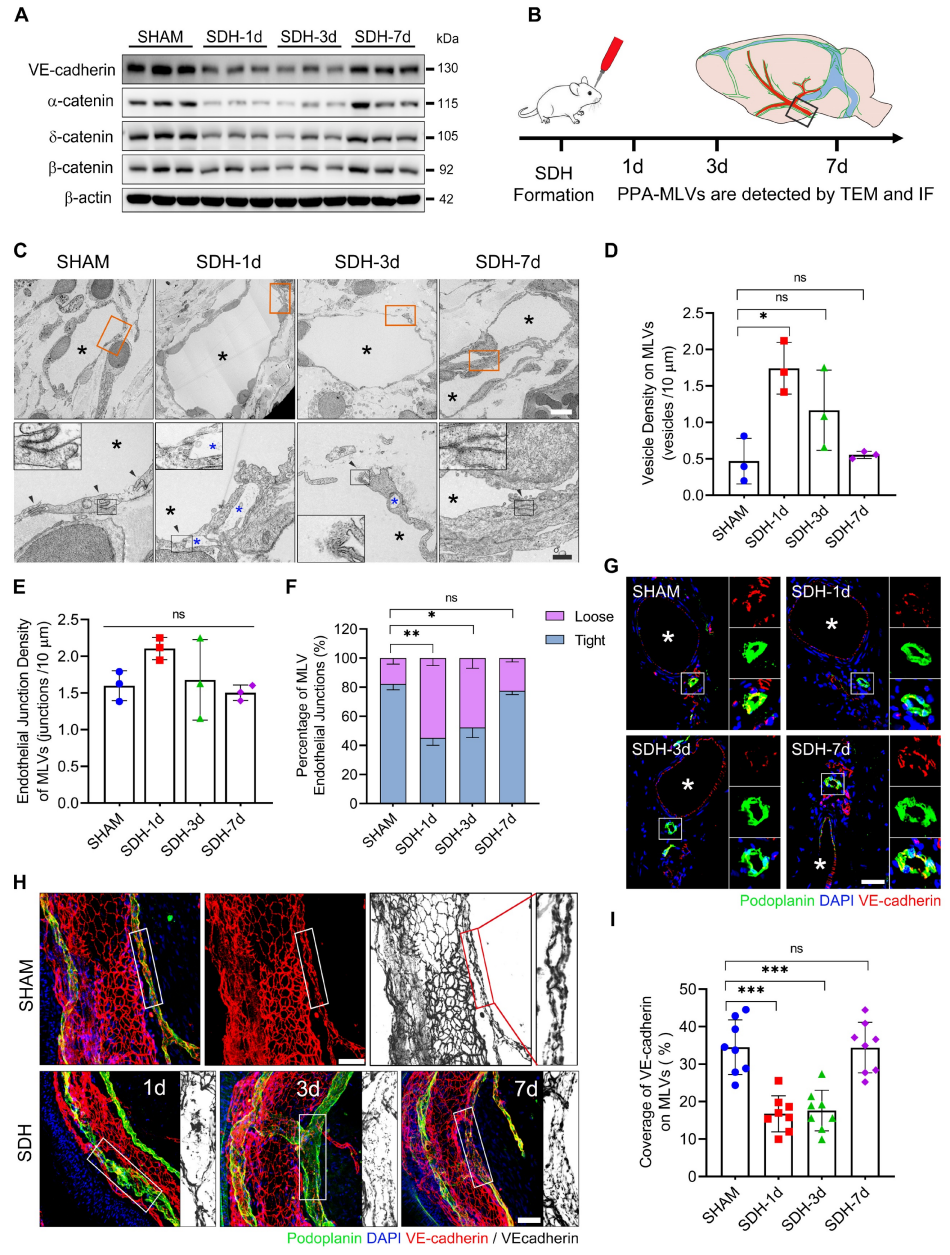
** SDH induces disruption of endothelial junctions within the basal MLVs. (A)** Western blot analysis of adherens junction structural protein expressions in the meninges following SDH. n = 3 rats per group. **(B)** Experimental protocol for the structural assessment of basal MLVs. **(C)** TEM images of ipsilateral basal MLVs. The second row represents a magnified view of the orange areas, including higher magnifications of endothelial junctions. Black arrows indicate lymphatic endothelial junctions, black asterisks represent lymphatic lumen, and blue asterisks represent interendothelial vesicles, which are frequently observed around the loose junctions. **(D-E)** The densities of interendothelial vesicles and endothelial junctions within the basal MLVs. n = 3 rats per group. **(F)** Quantifying the percentages of tight (see sham-type junction in **C**) or loose (see SDH-type junction in **C**) MLV endothelial junctions. n = 3 rats per group. **(G-H)** Immunostaining of VE-cadherin connections in the coronal and horizontal planes of ipsilateral basal MLVs, white asterisks represent PPA. **(I)** Quantifying the coverage percentage of VE-cadherin on the MLVs. n = 8 rats per group. Data are presented as means ± SD, statistical analysis with Welch's ANOVA followed by Dunnett T3 post hoc test in **D-F**, and one-way ANOVA followed by Dunnett post hoc test in **I**. * P < 0.05; ** P < 0.01; *** P < 0.001; ns, no significance. Scale bars: **C** 5 μm (upper) and 2 μm (lower); **G-H** 50 μm.

In addition to the decreased content of lymphatic proteins on the meninges, SDH also reduced the expression of VE-cadherin and three types of catenins (Figure 3A), which play vital roles in forming intercellular connections between lymphatic endothelial cells (LECs) 31. Using transmission electron microscopy (TEM), we identified a notable increase in the proportion of loose endothelial junctions within the ipsilateral basal MLVs, accompanied by a higher presence of interendothelial vesicles (Figure 3B-F). This pathological alteration has been suggested to compromise the intercellular junction integrity and potentially lead to lymph leakage 32. Additionally, immunostaining revealed a decreased density of VE-cadherin connections between the meningeal LECs (mLECs) on the first and third days following SDH (Figure 3G-I).

The disruption and subsequent restoration of the basal MLVs align with the dynamic changes in MLD efficiency. By the seventh day, the efficiency of MLD restored to normal levels. Although the lumen of the basal MLVs remained dilated, their endothelium exhibited tight connections. Furthermore, a significant increase in valve-related proteins FOXC2 and PROX1 expression was also observed. These findings were consistent between male and female rats, suggesting that the morphological alterations within the basal MLVs are accountable for the impaired MLD following SDH.

### SDH induces ERK1/2 dephosphorylation within the mLECs, leading to dysfunctional MLVs

**Figure 4:**
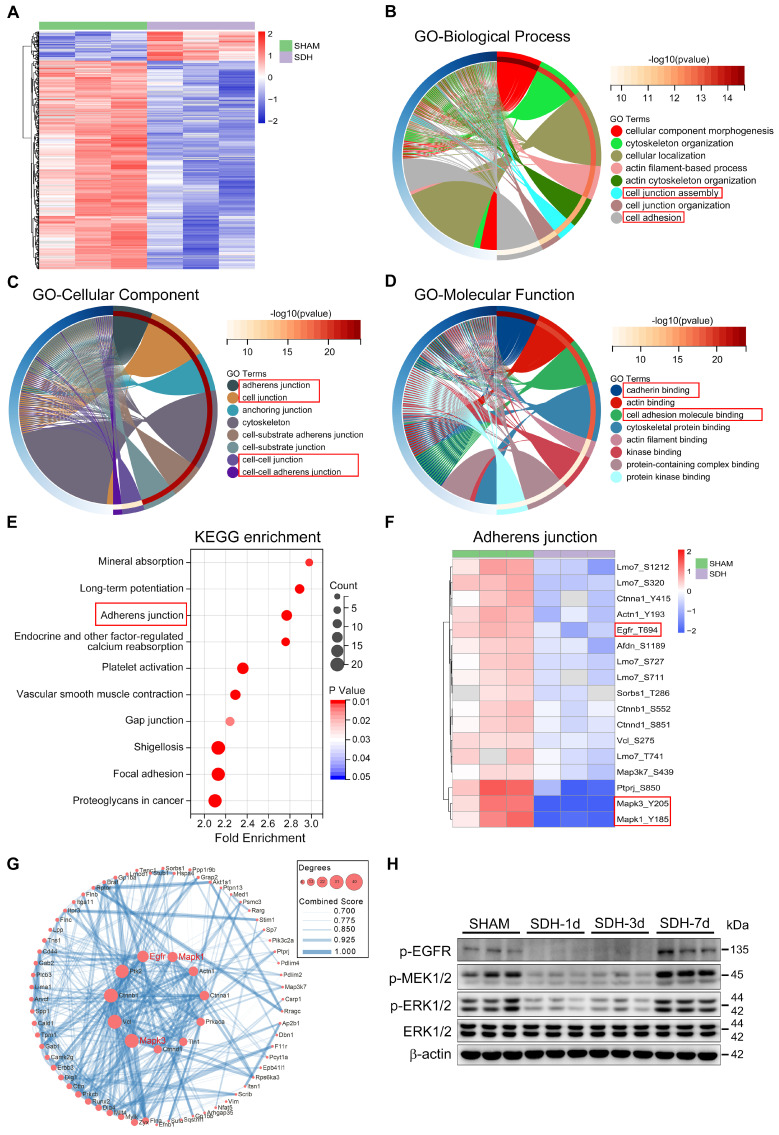
** Phosphoproteomic analysis of meninges following SDH. (A)** Heatmap showing relative phosphorylation levels of meningeal proteins in the SHAM and SDH samples. n = 3 rats per group. The significant GO enrichment terms in biological process **(B)**, cellular component **(C)**, and molecular function **(D)** of the altered proteins in the SHAM group compared to the SDH group. **(E)** The top ten KEGG enrichment pathways of the altered meningeal proteins following SDH. **(F)** Heatmap representation of the clustered proteins involved in adherens junctions and their specific phosphorylation sites. **(G)** Cytoscape analysis of protein-protein interactions. **(H)** Western blot analysis of phospho-EGFR, phospho-MEK1/2, phospho-ERK1/2, and total ERK1/2 expressions in the meninges. n = 3 rats per group.

To explore the underlying pathological mechanism behind the dysfunctional MLVs, we conducted phosphoproteomics analysis of meninges on the third day after SDH. There was a significant trend of dephosphorylation in meningeal proteins following SDH (Figure 4A). Specifically, 458 sites on 335 proteins exhibited dephosphorylation, while an increase in phosphorylation was observed at 64 sites in 56 proteins. Functional enrichment analysis based on the GO database revealed that the altered proteins predominantly localized in the adherens junction (AJ), playing crucial roles in cadherin binding and promoting the formation of cell junctions (Figure 4B-D). Another KEGG database analysis also exhibited a significant functional enrichment in the AJ signaling pathway (Figure 4E). These adhesion-related proteins, including Ctnna1, Ctnnb1, Ctnnd1, EGFR, and ERK1/2 (MAPK1/3), were all dephosphorylated by SDH. Among them, ERK1/2 exhibited the most pronounced dephosphorylation (Figure 4F). Furthermore, the altered proteins exhibited a close functional interaction network, with ERK1/2 playing a central role (Figure 4G). Significant dephosphorylation of ERK1/2 was detected at the acute phase following SDH, then gradual restoration over time. MEK1/2, the essential kinase of EKR1/2, and the upstream membrane receptor protein EGFR were also dephosphorylated by SDH (Figure 4H). Notably, the phosphorylation changes in the ERK1/2 signaling pathway were consistent with the dysfunctional MLVs.

**Figure 5:**
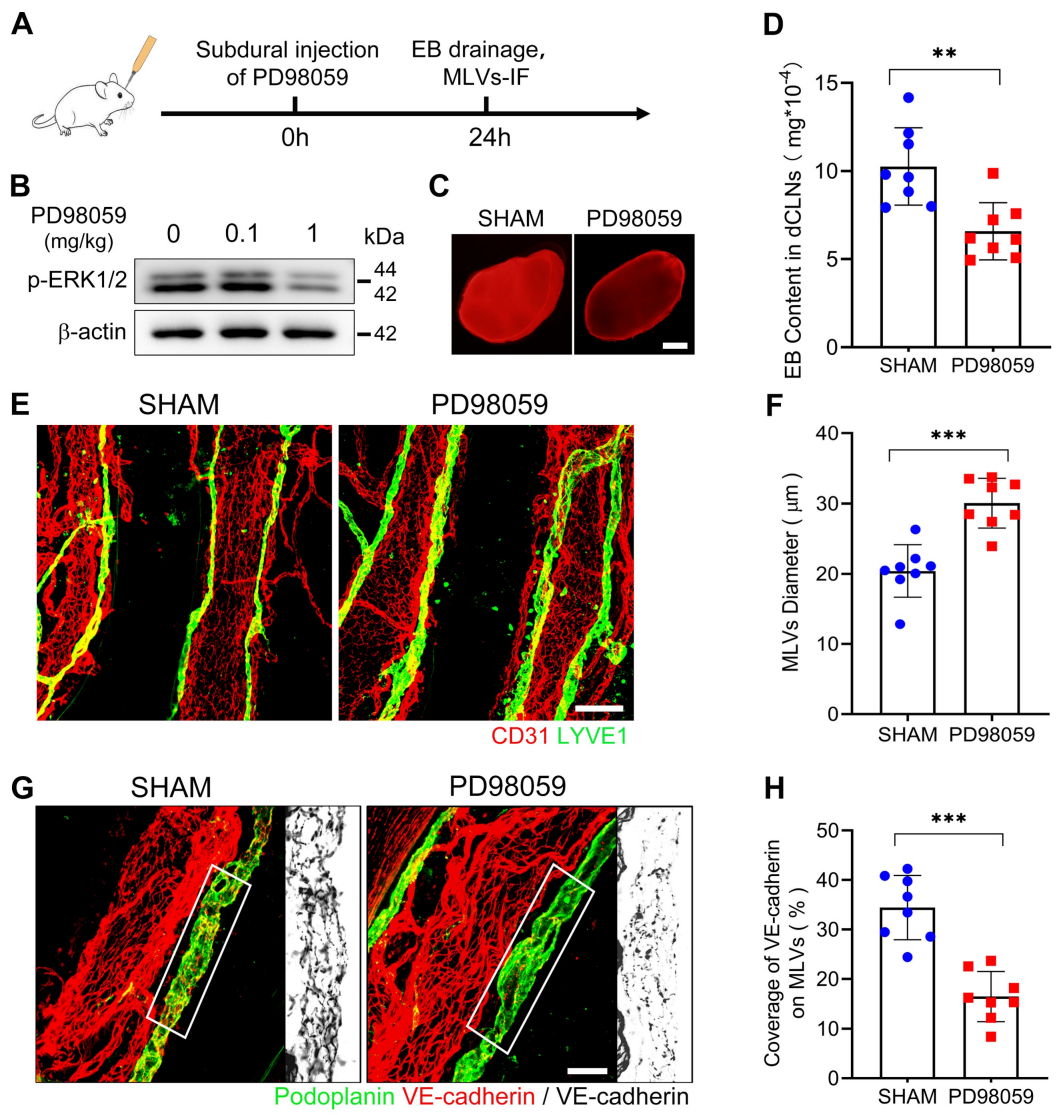
**ERK1/2 inactivation induces impaired MLD and disrupted basal MLVs. (A)** Schematic detailing of experimental procedures. **(B)** Western blot analysis of the phosphorylation level of ERK1/2 in the meninges. **(C-D)** Drainage efficiency of EB detected in the dCLNs. n = 8 rats per group. **(E)** Immunostaining of ipsilateral basal MLVs, and **(F)** quantification of the lymphatic lumen diameter. n = 8 rats per group. **(G)** Immunostaining of VE-cadherin connections in the basal MLVs, and** (H)** quantifying the coverage percentage of VE-cadherin on the lymphatic lumen. n = 8 rats per group. Data are presented as means ± SD, statistical analysis with two-tailed unpaired Student's *t* test in **D, F, H**. ** P < 0.01; *** P < 0.001. Scale bars: **C** 1 mm; **E** 100 μm; **G** 50 μm.

**Figure 6:**
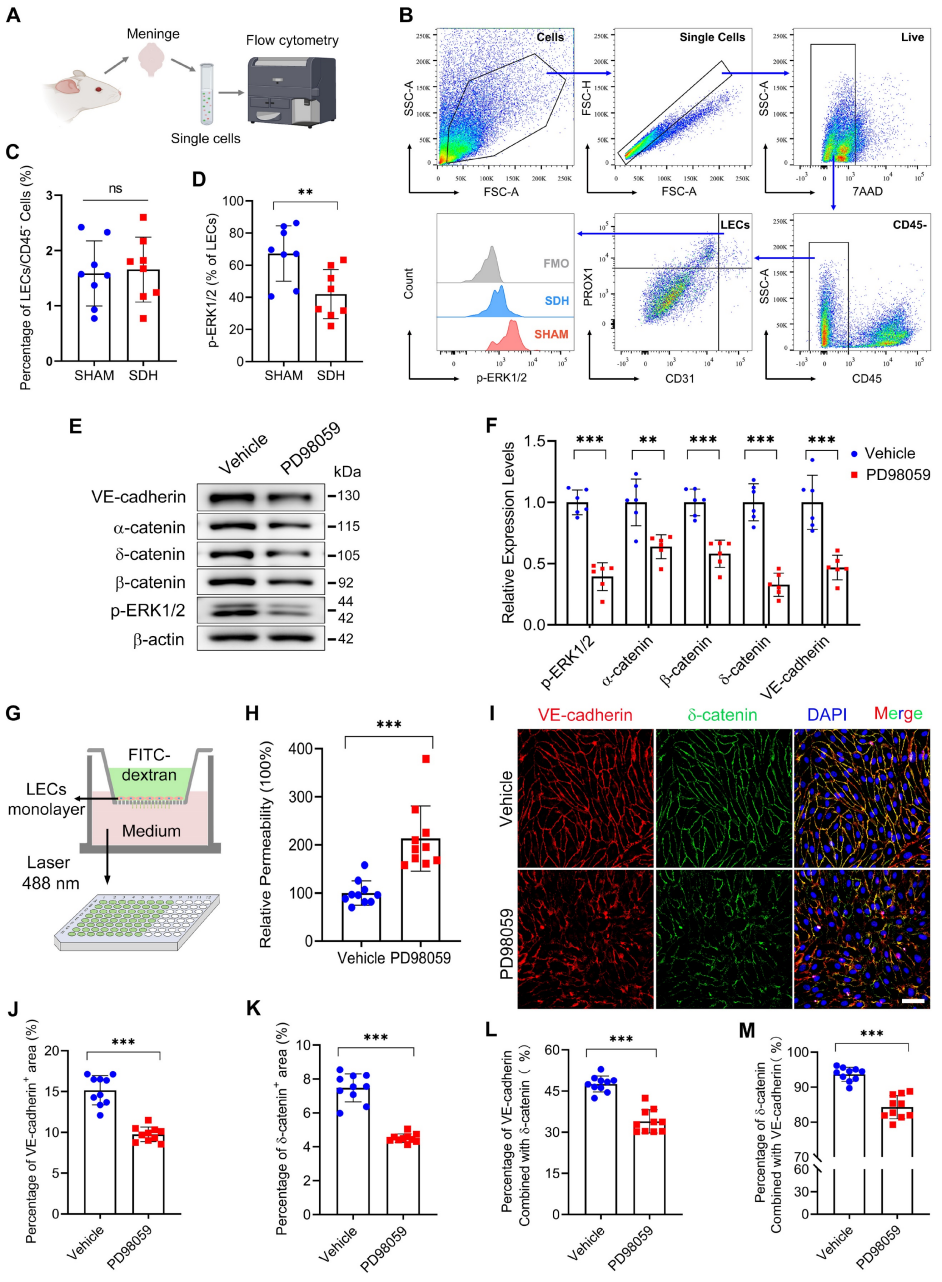
** ERK1/2 inactivation disrupts the endothelial junctions between LECs. (A-B)** Schematic diagram of meningeal flow cytometry procedure and gating strategy of mLECs. **(C)** Percentage of mLECs on the third day following SDH, and **(D)** flow cytometry analysis of ERK1/2 phosphorylation level in mLECs. n = 8 rats per group.** (E-F)** Western blot analysis of endothelial junction protein expressions in cultured LECs after ERK1/2 inhibition. n = 6 replicates per group. **(G)** Schematic diagram and **(H)** quantification of the cultured LEC monolayer permeability to 40 kDa FITC-dextran. n = 10 replicates per group.** (I)** Immunostaining of VE-cadherin and δ-catenin in cultured LECs. **(J-K)** Quantifying the percentage of VE-cadherin and δ-catenin positive area, and **(L-M)** the protein binding rate of VE-cadherin and δ-catenin. n = 10 replicates per group. Data are presented as means ± SD, statistical analysis with two-tailed unpaired Student's *t* test in **C-D, F, H, J-M**. ** P < 0.01; *** P < 0.001; ns, no significance. Scale bars: **I** 50 μm.

Next, to verify the impact of ERK1/2 dephosphorylation on MLD. We injected a specialized ERK1/2 kinase inhibitor PD98059 into the subdural space of rats and detected a dephosphorylation of ERK1/2 on the meninges 24h after injection (Figure 5A-B). After ERK1/2 inhibition, we re-injected EB into the subdural space and observed a diminished drainage of EB into the dCLNs (Figure 5C-D). Furthermore, there was a notable reduction in VE-cadherin connections within the basal MLVs, and the lumen of these lymphatic vessels exhibited dilation (Figure 5E-H). These findings indicate a connection between ERK1/2 activity and the function of MLVs. Flow cytometry further detected that ERK1/2 dephosphorylation occurred in the mLECs following SDH. However, the quantities of these mLECs are similar to the sham group (Figure 6A-D).

LECs are fundamental units of lymphatic vessels, playing a critical role in forming and maintaining the lymphatic lumen 33. ERK1/2 dephosphorylation occurring in LECs may contribute to pathological changes in lymphatic vessels. Subsequently, we inhibited the ERK1/2 signaling pathway in cultured human primary LECs. The dephosphorylation of ERK1/2 resulted in decreased expressions of adhesion-related proteins between the LECs (Figure 6E-F). Consistently, ERK1/2 inhibition resulted in increased permeability between cultured monolayer LECs. The added fluorescent dye was easier to leak through the lymphatic endothelial barrier, leading to higher concentrations of FITC-dextran detected in the lower chamber (Figure 6G-H). Immunostaining further exhibited decreased expressions of VE-cadherin and δ-catenin between the LECs (Figure 6I-K). In addition, the PD98059 treatment reduced the interactions between these two proteins, which form tight connections resembling screws and nuts between LECs (Figure 6L-M). The findings above reveal that SDH can cause ERK1/2 dephosphorylation in the mLECs, leading to disrupted endothelial junctions within the lymphatic lumen and dysfunctional MLVs.

### Atorvastatin protects the basal MLVs in SDH by preventing ERK1/2 dephosphorylation

**Figure 7:**
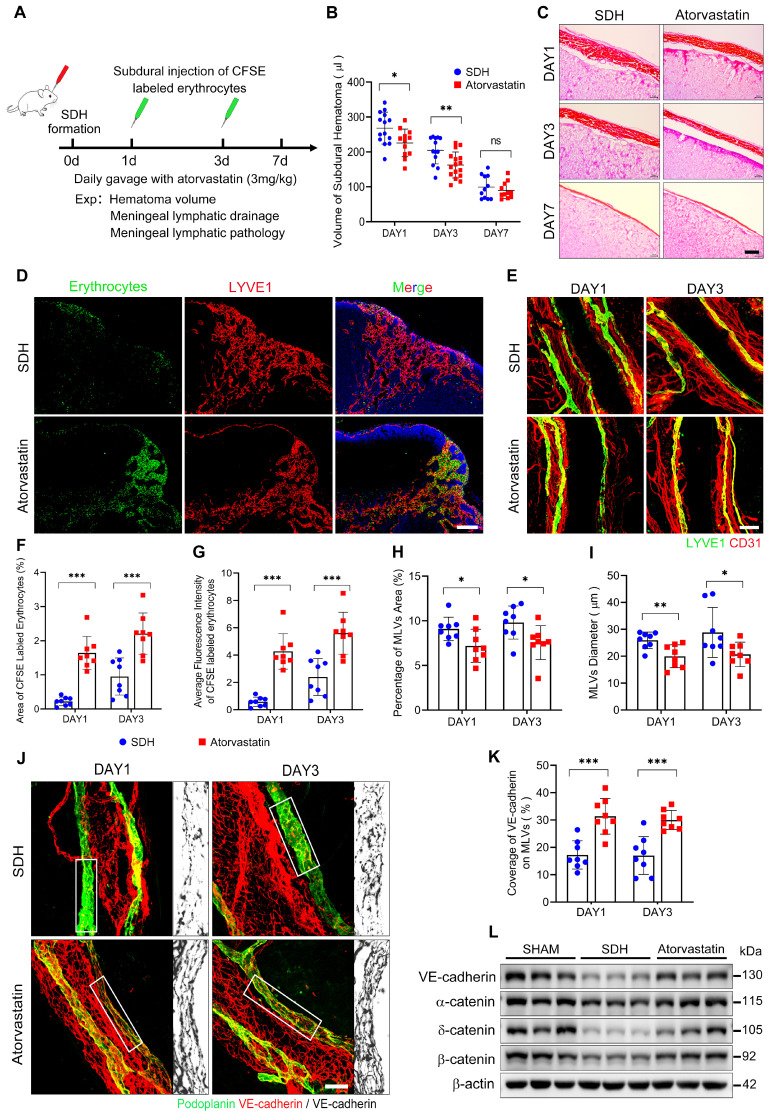
** Atorvastatin treatment improves MLD and prevents pathological alterations in basal MLVs. (A)** Schematic detailing of experimental procedures. **(B)** Quantification of the residual hematoma volumes and **(C)** H&E staining of SDH on the first, third, and seventh days. n = 11-16 rats per group. **(D)** Drainage efficiency of CFSE labeled erythrocytes detected in the ipsilateral dCLNs. **(E)** Immunostaining of the ipsilateral basal MLVs. The CFSE+ area percentage **(F)** and average fluorescence intensity of CFSE+ erythrocytes **(G)** in the ipsilateral dCLNs. n = 8 rats per group. Quantifying the coverage area **(H)** and lumen diameter **(I)** of the ipsilateral basal MLVs. n = 8 rats per group. **(J)** Immunostaining of VE-cadherin connections in the basal MLVs, and** (K)** quantifying the coverage percentage of VE-cadherin on the lymphatic lumen. n = 8 rats per group. **(L)** Western blot analysis of adherens junction structural protein expressions in the meninges. n = 3 rats per group. Data are presented as means ± SD, statistical analysis with two-tailed unpaired Student's *t* test in **B, F-I, K**. * P < 0.05; ** P < 0.01; *** P < 0.001; ns, no significance. Scale bars: **C** 40 μm; **D** 200 μm; **E** 100 μm; **J** 50 μm.

Treatment strategies to repair dysfunctional MLVs are meaningful to accelerate SDH clearance. Previous studies have shown a beneficial effect of atorvastatin on SDH treatment 23, 24, 34. However, it is uncertain whether atorvastatin can alleviate the pathological alterations in MLVs and facilitate MLD. Here, we treated SDH rats with atorvastatin by daily gavage (3 mg/kg) and evaluated its impact on MLD (Figure 7A). Atorvastatin administration accelerated hematoma absorption during the acute phase of SDH (Figure 7B-C), accompanied by MLD improvement. After injecting CFSE-labeled erythrocytes into the subdural space, the atorvastatin-treated group exhibited higher drainage and accumulation of CFSE+ erythrocytes in the dCLNs than the saline-treated control group (Figure 7D,F-G). Consistently, atorvastatin alleviated the pathological changes in basal MLVs.

Following treatment, the edematous expanded lymphatic lumen caused by hematoma was restored to normal diameter, accompanied by a tighter VE-cadherin connecting network (Figure 7E,H-K). Moreover, the treated group demonstrated higher expressions of adhesion-related proteins on the meninges (Figure 7L). The beneficial effects of atorvastatin on MLVs were achieved by preserving lymphatic structures rather than promoting lymphangiogenesis since the expressions of lymphatic proteins on the meninges did not increase following atorvastatin treatment, and the percentage of Ki67+ mLECs was similar to that in the saline-treated group (Figure S4A-C).

Previous phosphoproteomics analysis revealed a dephosphorylation trend in meningeal proteins following SDH. In contrast, atorvastatin treatment increased phosphorylation levels in multiple meningeal proteins compared to the control group (Figure S5A). One-third of these altered proteins (92/253, 36.4%) were also significantly affected by SDH (Figure S5B-C). Cluster analysis using the GO database demonstrated that these matched proteins were mainly located in the adherens junctions and cell junctions. The molecular functions of these proteins were mainly associated with cadherin binding (Figure S5D-E). All of these adhesion-related proteins were dephosphorylated following SDH, but atorvastatin treatment restored their phosphorylation, including ERK1 (Figure S5F). However, atorvastatin treatment specifically reactivated the ERK1/2 kinase MEK1/2 without affecting the upstream membrane receptor EGFR (Figure S5G).

**Figure 8:**
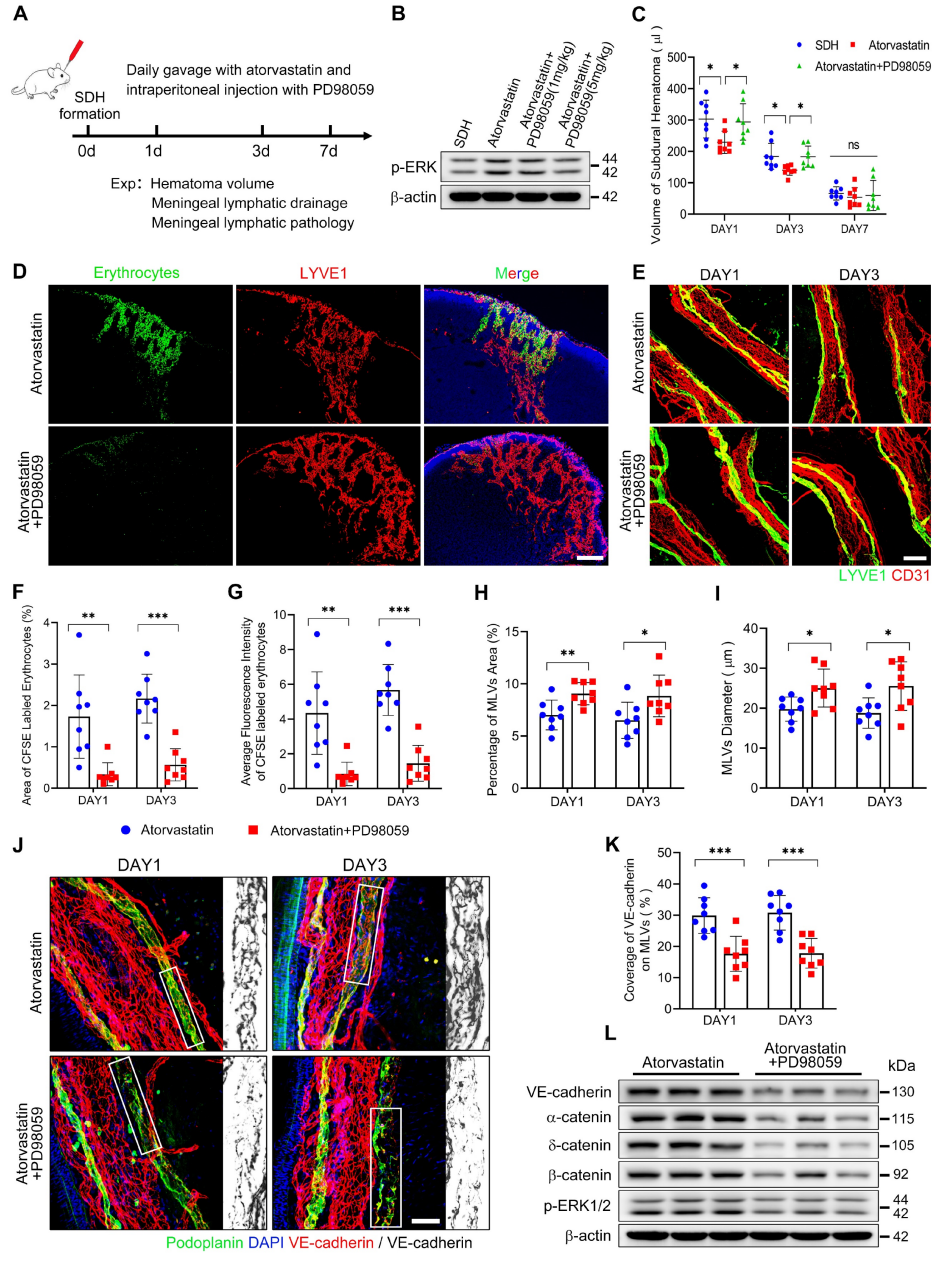
** The protecting effects of atorvastatin on MLVs are abolished upon inhibition of ERK1/2 signaling. (A)** Schematic detailing of experimental procedures. **(B)** Western blot analysis assessing the effect of PD98059 in inhibiting ERK1/2 signaling. **(C)** Quantification of the residual hematoma volumes. n = 8 rats per group. **(D)** Drainage efficiency of CFSE labeled erythrocytes detected in the ipsilateral dCLNs. **(E)** Immunostaining of the ipsilateral basal MLVs. The CFSE+ area percentage **(F)** and average fluorescence intensity of CFSE+ erythrocytes **(G)** in the ipsilateral dCLNs. n = 8 rats per group. Quantifying the coverage area **(H)** and lumen diameter **(I)** of the ipsilateral basal MLVs. n = 8 rats per group. **(J)** Immunostaining of VE-cadherin connections in the basal MLVs, and** (K)** quantifying the coverage percentage of VE-cadherin on the lymphatic lumen. n = 8 rats per group. **(L)** Western blot analysis of adherens junction structural protein expressions in the meninges. n = 3 rats per group. Data are presented as means ± SD, statistical analysis with one-way ANOVA followed by Tukey post-hoc test in **C** and two-tailed unpaired Student's *t* test in **F-I, K**. * P < 0.05; ** P < 0.01; *** P < 0.001; ns, no significance. Scale bars: **D** 200 μm; **E** 100 μm; **J** 50 μm.

The pathological alterations of MLVs following SDH were associated with ERK1/2 dephosphorylation. Atorvastatin treatment exhibited benefits in protecting MLVs and reactivating ERK1/2. Given our findings, we combined atorvastatin and PD98059 in SDH treatment (Figure 8A). Daily intraperitoneal injection of PD98059 (5 mg/kg) effectively blocked the reactivation of ERK1/2 by atorvastatin (Figure 8B). After ERK1/2 inhibition, atorvastatin did not accelerate the absorption of SDH. The volumes of residual hematoma in the combination-treated group were higher than the atorvastatin-treated group and similar to the control-treated group (Figure 8C). Furthermore, PD98059 hindered the improvement in MLD achieved by atorvastatin. In the combination-treated group, there was reduced drainage of subdurally injected CFSE+ erythrocytes into the dCLNs (Figure 8D,F-G). Consistently, we rediscovered edematous dilated MLVs on the basal meninges with disrupted endothelial connections within their lumen following combination treatment (Figure 8E,H-K). Moreover, PD98059 abolished the restoration of adhesion-related proteins expression on the meninges by atorvastatin (Figure 8L). The above findings demonstrate that atorvastatin can repair the dysfunctional MLVs following SDH through ERK1/2 activation.

## Discussion

The subdural space between the dura and arachnoid membrane is an independent cavity within the meninges 35. Following cerebral trauma, hemorrhages frequently accumulate in this space, giving rise to SDH 3. In this study, we investigate the pathophysiology of MLV system in experimental SDH rats and make three major discoveries: (ⅰ) Basal MLVs with abundant valves serve as hot spots for the drainage of subdural substances to the ipsilateral dCLNs, (ⅱ) SDH induces ERK1/2 dephosphorylation in the mLECs, leading to dysfunctional basal MLVs, (ⅲ) atorvastatin treatment restores basal MLVs and promotes SDH absorption via preventing ERK1/2 dephosphorylation.

Since the rediscovery of MLVs in 2015, they have been indicated to be involved in various neurological disorders, playing essential roles in facilitating cerebrospinal fluid (CSF) clearance and brain-derived antigen presentation 9-11, 13-19. This complex lymphatic network is exclusively detected within the dura mater and is separated from CSF by the arachnoid membrane 13, 26, 27. However, subdural substances, including SDH, directly contact MLVs without the arachnoid barrier, potentially leading to distinct drainage characteristics. Understanding the specific drainage process of subdural substances is essential to investigate the pathological mechanisms of impaired MLD following SDH.

In this study, we injected Gd into the subdural space and observed a significant drainage signal within the basal MLVs. Our finding aligns with prior studies reporting a higher accumulation of fluorescent dyes within basal MLVs after CSF injections 14, 26. This distinctive drainage process has been suggested to be correlated with morphological differences between dorsal and basal MLVs 26. The basal MLVs with abundant valves are classified as pre-collecting MLVs 26. These specific valve structures effectively prevent lymph reflux, enhancing lymphatic drainage efficiency 28, 36. However, despite the absence of typical valves in dorsal MLVs, a significant accumulation of Gd was also observed surrounding the TS. Previous findings have indicated that the dorsal MLVs adjacent to the TS are hotspots for CSF absorption 29, 37, and their deletion can result in impaired MLD 11, 16, 38. Our findings indicate the significance of both dorsal and basal MLVs in subdural substance clearance.

Prior research has also confirmed the involvement of cribriform plate in CSF clearance 39, 40. However, Gd injected into the subdural space was not detected within this location, suggesting a separation between the cribriform plate and the MLV system. Actually, the drainage of CSF observed in the cribriform plate is mainly derived from the olfactory nerve 40, 41. Despite being considered distinct pathways, both the cribriform plate and the MLV system ultimately drain CSF into the CLNs 14, 40. In our study, the subdurally injected dyes were mainly detected in the ipsilateral dCLNs, which have been indicated to be the primary drainage pool for MLD 13, 14. Overall, we demonstrate that MLVs exhibit similar drainage characteristics for subdural substances and CSF, while the cribriform plate, exclusively involved in CSF clearance, operates independently of MLD, advancing our understanding of the MLV system.

The observed powerful drainage effect of the basal MLVs and the ipsilateral drainage dominance phenomenon attracted our attention and were suggested to play a crucial role in SDH clearance. However, following SDH, the impaired MLD was mainly detected on the ipsilateral side, accompanied by significant morphological alterations in the basal pre-collecting MLVs exhibiting dilated lumen and disrupted endothelial connections. These dilated MLVs were not a manifestation of lymphangiogenesis, as we did not observe an increased proliferation of mLECs. The pathologically dilated pre-collecting lymphatics in the peripheral lymphatic system are defined as lymphedema 31. Lymph transport is frequently impaired within these vessels due to inadequate valve function. In our study, SDH resulted in decreased expression of meningeal lymphatic valve proteins FOXC2 and PROX1, further indicating valvular inadequacy in these dilated basal MLVs. Notably, the dysfunctional MLVs observed in TBI, neurotropic viral infections, and aged mice are also accompanied by decreased expressions of FOXC2 and PROX1 26, 42, 43. Besides the defective valves, we also found disrupted connections between the mLECs in the basal MLVs, which have also been reported in aging and Parkinson's disease 18, 26. The severe destruction of endothelial junctions in MLVs can lead to impaired MLD, as these junctions play a crucial role in maintaining lymphatic drainage 31, 44.

Dysfunctional MLVs have been demonstrated in various neurological disorders, exhibiting diverse pathological alterations 15, 16, 18, 19, 26, 42, 45. The diameters of MLVs are most described but observed with opposite changes. Dilated MLVs are observed in TBI and viral infections 15, 42, while narrower lymphatic lumens are identified in AD and depression 16, 45. However, these studies primarily focus on the dorsal MLVs, which are absent of lymphatic valves. Their drainage function is exempt from valvular inadequacy and remains unimpaired following dilation. Instead, dilated dorsal MLVs have been suggested to accelerate MLD and facilitate neurotoxic substances clearance 16, 45, 46. Therefore, the impaired MLD observed following TBI, viral infections, and SDH is more likely related to the dysfunctional valves within the basal MLVs. Studies also detect increased apoptosis in the mLECs following viral infection and SAH, and inhibiting the apoptosis pathway improves MLD in SAH 42, 47. Notably, the mice with depression also exhibit decreased mLECs 45, whereas this change was not detected in SDH rats. Apart from the pathological alterations of MLVs, increased ICP has also been indicated to be involved in MLV dysfunction 15, 29. However, in our study, the increased ICP following subdural injection rapidly resolved within hours, while the impaired MLD persisted for days, suggesting the involvement of additional pathological alterations.

There is no dispute that endothelial junctions are essential for maintaining the integrity of lymphatic vessels 48, and their disruption has been shown to impair MLD 26, 49. Here, we indicated that the disrupted connections between mLECs following SDH were associated with ERK1/2 dephosphorylation. ERK1/2 are dual-specificity kinases involved in various biological processes, encompassing transcription, proliferation, and cell adhesion 50. They have been reported to participate in the formation and maintenance of AJs 51. Furthermore, the inactivation of ERK1/2 has been demonstrated to disrupt cell-cell junctions, characterized by decreased expressions of VE-cadherin 52, 53, a transmembrane adherens protein that forms AJs with intracellular catenins to maintain lymphatic vessel integrity 44, 54. In our study, SDH resulted in significant dephosphorylation of ERK1/2 within the mLECs, accompanied by dilated lumen and disrupted endothelial connections. We further demonstrated that inactivation of ERK1/2 could decrease VE-cadherin and catenins expression between the LECs and disassemble their binding, resulting in endothelial barrier failure and impaired MLD. The ERK1/2 signaling has been confirmed to be involved in lymphangiogenesis 55, 56. However, its involvement in maintaining lymphatic endothelial junctions is demonstrated for the first time. Notably, ERK1/2 inactivation can lead to dysfunctional MLVs, suggesting an essential role of ERK1/2 signaling in supporting MLV function.

As a downstream component in the Ras-Raf-MEK-ERK signaling cascade, ERK1/2 are primarily activated by their unique kinase MEK1/2 50. Following SDH, both MEK1/2 and ERK1/2 exhibited rapid dephosphorylation. This simultaneous alteration was suggested to be linked to EGFR activity, which exhibited synchronized changes with ERK1/2 and MEK1/2. EGFR can undergo autophosphorylation upon ligand binding, activating downstream Ras-Raf-MEK-ERK cascades 57. However, the activation of ERK1/2 signaling is a complex biological process. Following SDH, the decreased expression of VEGFR3 and catenins may also be accountable to ERK1/2 dephosphorylation, as these proteins have been reported to be involved in the activation of ERK1/2 signaling 58, 59. Moreover, the dephosphorylated meningeal proteins detected in SDH rats exhibited functional enrichment in kinase binding, indicating potential disruptions in protein kinase connections and restricted signal transduction. These findings may collectively contribute to the dephosphorylation of ERK1/2 observed in SDH, and the detailed mechanisms remain to be studied.

Based on the above findings, repairing the dysfunctional MLVs could be an effective treatment for SDH. Currently, atorvastatin is utilized in CSDH treatment and has been shown to accelerate hematoma absorption via alleviating subdural inflammation 20, 21, 24. In this study, we newly identified a protective effect of atorvastatin on MLVs in SDH rats. Atorvastatin treatment increased the phosphorylation of ERK1/2 and protected endothelial junctions between the mLECs. Nevertheless, when ERK1/2 signaling was inhibited, the beneficial effects of atorvastatin on MLVs were nullified, indicating that atorvastatin might protect MLVs from SDH-induced damage by sustaining ERK1/2 activation. Prior research has indicated that atorvastatin can increase the activity of ERK1/2 by enhancing MEK1/2 phosphorylation 60. Correspondingly, atorvastatin treatment restored the inactivated MEK1/2 following SDH but had no discernible impact on the upstream receptor EGFR. The specific target of atorvastatin in regulating the MEK/ERK signaling pathway needs further investigation.

Statin treatment has been used in various neurological disorders and has demonstrated beneficial effects through its lipid-lowering, anti-inflammatory, antioxidant, and endothelial protection properties. To the best of our knowledge, our study is the first to suggest a potential role for atorvastatin in regulating MLD. Interestingly, this effect may have been implied in previous studies, which indicate that atorvastatin can reduce the formation of neurofibrillary tangles and β-amyloid expression in AD 61, 62. The clearance of these neurotoxic proteins is closely related on MLV function 16. Therefore, further investigation is needed to explore the effect of atorvastatin on MLV system and its potential association with neurological improvement.

The major finding of this work highlights that inactivation of ERK1/2 signaling mediates the dysfunctional MLVs in SDH rats. However, the specific mechanisms for ERK1/2 dephosphorylation following SDH require further investigation. Moreover, ERK1/2 inactivation following SDH or PD98059 administration was not unique to mLECs. Whether ERK1/2 dephosphorylation occurs in other cell types could indirectly affect MLVs or influence hematoma clearance remains to be studied. Further, in addition to the inactivation of ERK1/2 signaling, SDH also affected the activity of other adhesion-related proteins, which should also be considered potential contributors to the pathological MLVs. Finally, we only used adult rats in this study. Since CSDH incidence is age-related 63, further research is needed to investigate the impact of age-related MLV dysfunction on CSDH in elderly rats and whether atorvastatin treatment can preserve MLV integrity in older rodents.

In summary, for the first time, we identified the specific characteristics of MLD in subdural substance clearance and revealed a landscape of structural and molecular alterations of MLVs following SDH. We discovered significant dilated basal MLVs with disrupted endothelial junctions, leading to impaired MLD. Moreover, these pathological changes in the basal MLVs were indicated to be associated with ERK1/2 inactivation. These findings complement the pathology of SDH and suggest a relationship between the pathological changes in basal MLVs and the impairment of MLD. We also discovered an additional therapeutic effect of atorvastatin in stabilizing the MLV structures and improving MLD. It will be worth examining in the future whether the protective effect of atorvastatin on MLVs can be a potential intervention for other neurological disorders.

## Methods

### Animals

Female and male Sprague‒Dawley rats (9-10 weeks old and weighing 300-320 g; HFK Bioscience Co., Ltd., Beijing, China) were randomly selected and housed in a temperature-controlled room (20 ± 2 °C) in a 12-h light/dark cycle, with ad libitum access to food and water. All experimental procedures were approved by the Tianjin Medical University Animal Ethics Committee (Tianjin, China) and conducted according to the Animal Research: Reporting of *In vivo* Experiments (ARRIVE) guidelines.

### *In vitro* LEC culture

Primary human LECs (iCell Bioscience Inc., cat no. HUM-iCell-i008, Shanghai, China) were cultured in endothelial cell medium (ECM) (iCell Bioscience Inc., cat no. PriMed-iCell-002) supplemented with 5% fetal bovine serum and incubated in a humidified incubator at 37 °C with 5% CO_2_. All LECs used in our study were obtained from passages 4-5.

### SDH models formation and atorvastatin treatment

As described in the previous study, we established an SDH rat model by subdural injection of autologous blood 10. Rats were anesthetized with isoflurane (5% induction, 2% maintenance) and prepared by shaving the hair and disinfecting the scalp. A 1-cm incision along the midline exposed the dorsal skull, and a 1.5 mm diameter, 1 mm deep bone window was drilled 3 mm to the right of the bregma. The dura mater was carefully torn using microscopic tweezers, and 400 µL of autologous blood from the femoral vein was injected into the subdural space at a rate of 50 µL/min using a trace injection pump (RWD Bioscience Co., Ltd., Shenzhen, China) equipped with 14G indwelling needles. The indwelling needle was left in place for 10 min to prevent blood leakage before removal. The bone window was sealed with medical glue, and the scalp incision was disinfected, sutured, and postoperative pain was alleviated with intraperitoneal injection of ibuprofen (10 mg/kg). A sham group underwent the same surgical procedure without blood injection, and all surgeries were performed using sterile medical instruments.

For atorvastatin treatment, rats received daily intragastric administration of atorvastatin (Pfizer, US; 3 mg/kg, dissolved in saline). This dosage was consistent with our previous studies 23, 34. The control group received an equal volume of saline.

### 9.4 T MRI scan of the head and neck

Rats were anesthetized with isoflurane (5% induction, 2% maintenance) and then examined using a 9.4 T MRI scanner (Bruker BioSpec 94/30) with a linearly polarized coil (40-mm inner diameter) and ParaVision 6.0.1 software. Gd was diluted in 0.9% NaCl at a concentration of 5 mM. Then, 200 µL solution was injected into the subdural space at 50 µL/min using a trace injection pump equipped with 14G indwelling needles. Contrast-enhanced imaging was achieved with a 3D T1 echo sequence with the following parameters: repetition time, 800.0 ms; echo time, 5.8 ms; rare factor, 2; field of view, 40.0 mm **×** 40.0 mm **×** 0.8 mm; average, 2; and scan time, 3 min, 24 s, 800 ms. A total of 22 or 40 consecutive slices were acquired for whole-brain and neck coverage, respectively.

### Functional assessment of MLD

MLD efficiency was assessed using two methods. First, 50 µL of 2% EB dye (Sigma‒Aldrich, cat no. E2129) was injected into the subdural space. After 30 min, rats were anesthetized with 5% isoflurane, followed by euthanasia through cervical dislocation. Subsequently, they were transcardially perfused with ice cold phosphate-buffered saline (PBS), and the CLNs were harvested. The EB dye within the CLNs was extracted with formamide and quantified using a microplate reader (Thermo Fisher Scientific, US).

To better assess the drainage efficiency of MLVs for hematomas, erythrocytes labeled with CFSE (eBioscience, cat no. 65-0850-84) to emit green fluorescence were injected into the subdural space. The efficiency of MLD was determined by calculating the CFSE-labeled erythrocytes collected in the CLNs. Initially, 1 mL of blood was collected from anesthetized rats and mixed with sterile PBS containing 5% BSA. The samples were centrifuged, and the plasma was discarded. The erythrocytes were resuspended in 1 mL of 5% BSA-PBS, followed by another centrifugation step. After two rounds of suspension and centrifugation, the erythrocytes were diluted to a concentration of 1 × 10^6 cells/mL using sterile PBS. Then, CFSE (20 μM/mL) was added to the suspension and incubated for 20 min at 37 °C. After incubation, the suspension was diluted 1:4 with 5% BSA-PBS and incubated on ice for 5 min to terminate the reaction. The erythrocytes were washed three times with 5% BSA-PBS, resuspended in 100 µL of sterile PBS, and immediately injected into the subdural space. After 30 min, the CLNs were harvested, and three 10-μm-thick frozen sections were prepared from the broadest section of each CLN with 0.5 mm intervals. The sections were imaged using an Olympus IX73 microscope (Japan) with a 10× objective and analyzed using ImageJ software (NIH) to obtain the mean value. The final results were recorded in Microsoft Excel.

### ICP monitoring

Rats were anesthetized with isoflurane, and their scalps were shaved and disinfected. A 1.5 mm diameter bone window was drilled 3 mm to the right of the bregma for subdural injection, while a contralateral 0.5 mm diameter bone window was drilled for ICP monitoring. A pressure sensor catheter (transonic, cat no. FTH-1211B- 0018, USA) was then inserted perpendicularly into the left parenchyma at a depth of 1 mm to record ICP continuously (Figure S2G). Following stabilization of the ICP signal, a 30-minute continuous recording began, with subdural injection starting at 5 min and lasting for 8 min. After 24 h, rats were re-anesthetized for a 5-minute ICP monitoring session, and the data were averaged for statistical analysis.

### Meninges immunofluorescence

Following euthanasia, rats were transcardially perfused with ice cold PBS to remove circulating blood. Next, we used a surgical scalpel to create a midline incision along the sagittal suture, weakening the connections between the bilateral skulls. Subsequently, at the edge of the foramen magnum, the occipital bone was firmly held with a needle holder, leveraged outward, and removed. The identical procedure was then employed to individually secure, lever open, and extract the bilateral parietal and frontal bones. Then, the dorsal meninges attached to the brain were cut with micro-scissors along the temporal bone. After removing the brain, the basal meninges were carefully stripped from the basal skull using a nerve root retractor. All the meninges were immediately fixed with 4% PFA overnight at 4 °C before staining.

After fixation, the residual PFA was washed away. The meninges were incubated with 5% BSA, 0.3% Triton X-100, and 0.05% Tween-20 in PBS at room temperature (RT) for 2 h, followed by incubation with appropriate dilutions of primary antibodies overnight at 4 °C in PBS supplemented with 3% BSA and 0.5% Triton X-100. After washing with PBS three times at RT, the meninges were incubated with secondary antibodies for 2 h at RT in PBS. Finally, the meninges were mounted with coverslips and imaged using a confocal fluorescence microscope (Olympus, FluoView 1200, Japan) with a 20× or 40× objective. All images were acquired at a resolution of 1024 × 1024 pixels and a z-step of 5 µm. The diameters of basal MLVs were resulted by tracing the region of interest (ROI) of lymphatic vessels along the PPA and measuring the vessel width at 40-µm intervals using ImageJ software (NIH). All data were recorded in Microsoft Excel, and the mean value was calculated as the final result.

### Electron microscopy

After euthanasia, rats were not perfused to preserve erythrocytes within the blood vessels, enabling the distinction between meningeal blood vessels and lymphatic vessels. The meninges were harvested as mentioned above and fixed in 2.5% glutaraldehyde and 2% PFA in 0.1 M sodium cacodylate buffer for 24 h at 4 °C. Then, the samples were fixed in 2% osmium tetroxide in 0.1 M sodium cacodylate buffer. Next, the meninges were dehydrated by incubation with a series of graded ethanol solutions, infiltrated with epoxy resin, and polymerized for 24 h at 70 °C. Ultrathin sections (80 nm) were cut, stained with 1% uranyl acetate and 1% lead citrate, and imaged using TEM (HT7800; HITACHI, Japan).

### H&E staining

To obtain coronal sections of rat brains with intact meninges, we harvested brains without skull removal and fixed them in 4% PFA for 2 days at 4 °C. The skulls were then thinned using an electric dental drill, and the remaining bone was removed with micro-scissors and tweezers. Brains with intact meninges were used to prepare 10-μm-thick frozen sections and stained using an H&E staining kit (Solarbio, cat no. G1120) following the manufacturer's instructions.

### Quantification of hematoma volumes

As described in previous studies, hemoglobin levels were quantified to determine the residual hematoma volume 10. Following euthanasia, rats were transcardially perfused with ice cold PBS to remove circulating blood. The collected meninges with hematomas were ground, ultrasonicated, and centrifuged (13000 RPM, 30 min) in 1 mL of PBS. A 0.4 mL aliquot of the resulting supernatant was mixed with 0.6 mL of Drabkin's reagent (Sigma-Aldrich, cat no. D5941). After a 15-minute reaction at RT, 200 µL of the mixture was added to a 96-well plate. The absorbance of each well was measured at a wavelength of 540 nm using a microplate reader. Autologous blood was also processed similarly to establish a standard curve with eight concentrations ranging from 0 to 400 µL. The residual SDH volume was calculated based on the absorbance values using Excel.

### Phosphorylated proteomics

Phosphorylated 4D label-free quantitative (LFQ) proteomics and analysis were conducted by Jingjie PTM BioLab Co. Ltd. (China). The entire meninges were harvested and ground into powder using liquid nitrogen. The resulting powder was generated into phosphopeptide through a lysis process using specialized buffers. The enriched phosphopeptides, obtained through UPLC separation, were subjected to ionization and analysis using tims-TOF Pro mass spectrometry (Bruker, Germany). The acquired MS/MS data were processed using the MaxQuant search engine (v.1.6.15.0). Tandem mass spectra were searched against the Rattus norvegicus Uniprot database. A mass tolerance of 20 ppm was set for precursor ions in the first search, main search, and fragment ions. Phosphorylation on Ser/Thr/Tyr residues was designated as a variable modification. The LFQ method was employed for quantitative analysis, while the false discovery rate (FDR) thresholds for protein, peptide, and modification sites were conservatively set at 1%.

### Western Blot

Proteins were extracted from rat meninges and primary human LECs following our previously published protocol. A phosphatase inhibitor (Invitrogen, cat no. A32957) was added to the lysis buffer to facilitate the analysis of phosphorylated proteins. The proteins were separated by 10% SDS-PAGE and transferred to PVDF membranes. After blocking with 5% BSA, the PVDF membranes were incubated with primary antibodies overnight at 4 °C on a shaker. Following thorough washing, the membranes were incubated with secondary antibodies for 1 h at RT and visualized using a ChemiDoc imaging system (Bio-Rad).

### Flow cytometry

After pericardial perfusion with ice cold PBS, the meninges were harvested and cut into small pieces. Erythrocytes were removed using red blood cell lysis buffer (Solarbio, cat no. R1010). After washing with PBS, collagenase IV (Solarbio, cat no. C8160) was used to lyse the meninges at 37 ℃ for 30 min. After antigen blocking with 1% BSA-PBS, resuspended cells were incubated with surface markers at 4 ℃ for 30 min. After staining, cells were fixed using a fixation/ permeabilization buffer (eBioscience, cat no. 00-5123-43) at RT for 20 min, subsequently incubated with intracellular antibodies at 4 ℃ for 30min. Finally, indirectly labeled primary antibodies (CD31 and p-ERK1/2) were stained with fluorescent secondary antibodies at 4 ℃ for 30 min. After staining, samples were analyzed using BD LSRFortessa flow cytometer (BD Bioscience), and data were analyzed with FlowJo 7.6 software.

### Primary human LEC permeability assay

Primary human LECs were seeded on Transwell filters (LABSELECT 14211; 12 mm, 0.4-μm-pore polycarbonate membrane) and grew to confluence. Following 2 h serum-free starvation, LECs were treated with the ERK1/2 inhibitor PD98059 (MCE, HY-12028, 10 μM in ECM-basal) for 20 h, and the control treatment consisted of PBS without PD98059. After incubation, 200 μL of FITC-dextran (40 kDa; Sigma-Aldrich, 1 mg/mL diluted using ECM-basal) was added to the upper chamber of the Transwells, allowing for trans-cellular diffusion over 1 h. The fluorescence intensity in the lower chamber was measured using a microplate reader, and the integrity of the monolayer was confirmed through immunofluorescence staining.

### Statistical analysis

The sample size in this study was similar to those reported in previous studies 11, 16, 18, 26, 42. Animals were randomly grouped; all experiments in this study were repeated more than two times, and all analyses were performed by investigators blinded to the experimental group. The Shapiro-Wilk test was used to assess the normal distribution of the data, and a two-tailed unpaired Student's *t* test was used to compare data from two independent groups. One-way analysis of variance (ANOVA) followed by Dunnett or Tukey post-hoc test was used to compare data from three or more independent groups with similar variances, and Welch's ANOVA followed by the Dunnett T3 post-hoc test was used to compare groups with unequal variances. Statistical analysis was performed using GraphPad Prism 8.0 software, and statistical significance was set at P < 0.05. All data were expressed as the mean ± standard deviation (SD).

## Supplementary Material

Supplementary figures and table.

## Abbreviations

MLVs: meningeal lymphatic vessels
SDH: subdural hematoma
CSDH: chronic subdural hematoma
MLD: meningeal lymphatic drainage
Gd: Gadopentetate D
EB: evans blue
LECs: lymphatic endothelial cells
mLECs: meningeal lymphatic endothelial cells
AJ: adherens junction
dCLNs: deep cervical lymph nodes
sCLNs: superficial cervical lymph nodes
TBI: traumatic brain injury
AD: Alzheimer's disease
SAH: subarachnoid hemorrhage
SSS: superior sagittal sinus
TS: transverse sinus
MMA: middle meningeal artery
PPA: pterygopalatine artery
SS: sigmoid sinus
PSS: petrosquamosal sinus
MRI: magnetic resonance imaging
TEM: transmission electron microscopy
CSF: cerebrospinal fluid
ICP: intracranial pressure
PBS: phosphate-buffered saline
PFA: paraformaldehyde
RT: room temperature
ECM: endothelial cell medium
ANOVA: one-way analysis of variance
SD: standard deviation
