# Supplementary material for: Inactivation of ERK1/2 signaling mediates dysfunction of basal meningeal lymphatic vessels in experimental subdural hematoma
Source: Theranostics. 2024 Jan 1;14(1):304–23. doi: 10.7150/thno.87633 (PMC10750213; doi:10.7150/thno.87633)
Supplement: Supplementary file 1 — Supplementary figures and table. [file thnov14p0304s1.pdf]

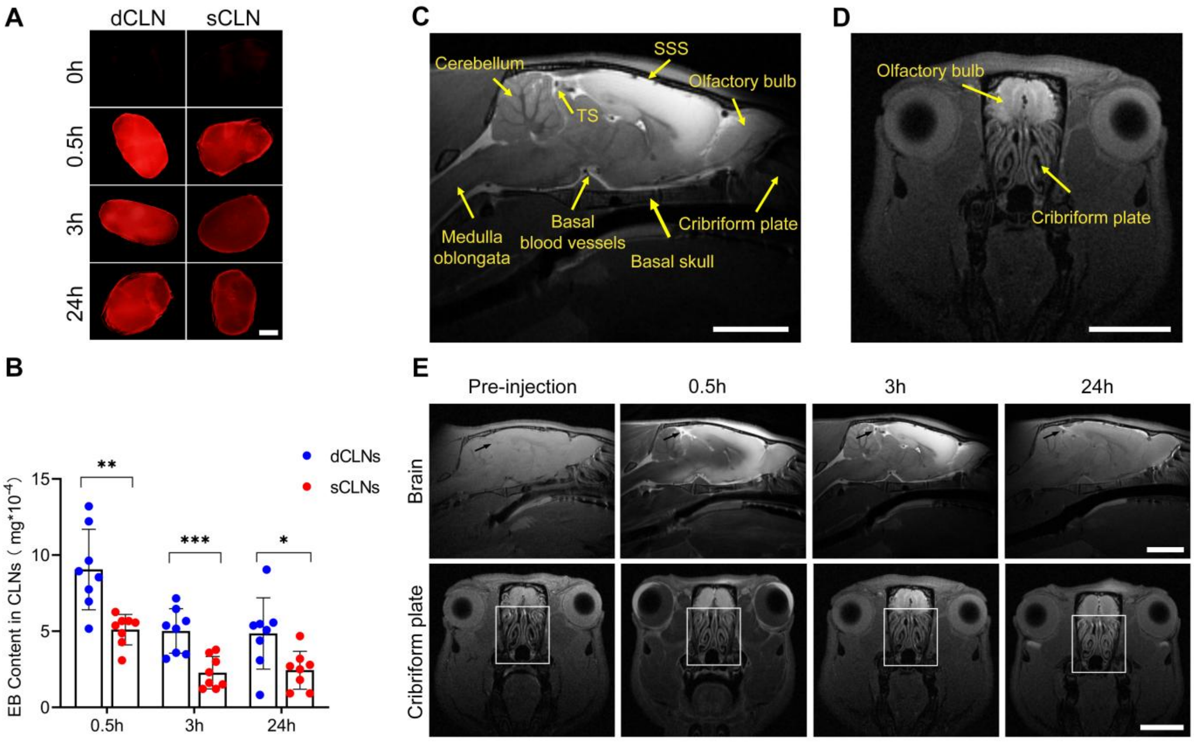

Figure S1. Subdural substances preferentially drain to the dCLNs without entering the cribriform plate. **(A)** The drainage efficiency of subdurally injected EB in the sCLNs and dCLNs. **(B)** Quantifying the EB content in the sCLNs and dCLNs. n = 8 rats per group. **(C-D)** Schematic diagrams of the sagittal brain and cribriform plate. **(E)** Gd drainage process observed in the brain and cribriform plate. Black arrows indicate TS; white rectangles indicate cribriform plate. Data are presented as means ± SD, statistical analysis with two-tailed unpaired Student's *t* test in **B**. * $P < 0.05$; ** $P < 0.01$; *** $P < 0.001$. Scale bars: **A** 1mm; **C-E** 1cm.

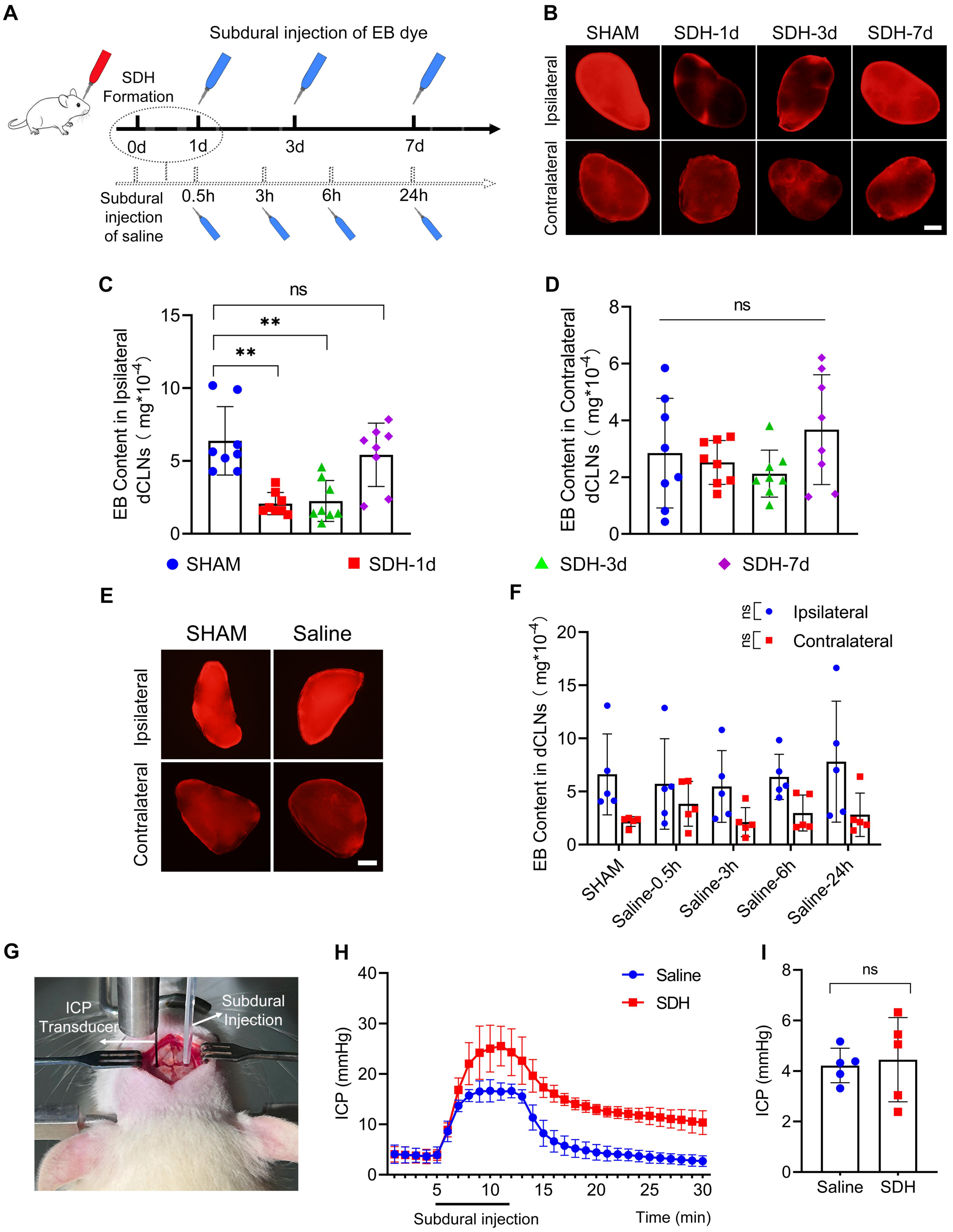

Figure S2. Impaired MLD following SDH primarily occurs on the ipsilateral side without increased ICP. **(A)** Schematic detailing of experimental procedures. **(B)** The drainage efficiency of subdurally injected EB in the bilateral dCLNs. **(C-D)** Quantifying the EB content in the ipsilateral dCLNs and the contralateral dCLNs. n = 8 rats per group. **(E-F)** Assessing the drainage efficiency of EB in the bilateral dCLNs at various time points after subdural saline injection. n = 5 rats per group. **(G)** Schematic diagram of ICP monitoring. **(H)** The time course of ICP before and after subdural injection of 400μL autologous blood or saline. Injection starts at

5-min and completes at 13-min. n = 5 rats per group. **(I)** ICP monitoring at 24-hour following subdural injection. n = 5 rats per group. Data are presented as means ± SD, statistical analysis with one-way ANOVA followed by Dunnett post hoc test in **C-D, F** and two-tailed unpaired Student's *t* test in **I**. ** P < 0.01; ns, no significance. Scale bars: **B, E** 1mm.

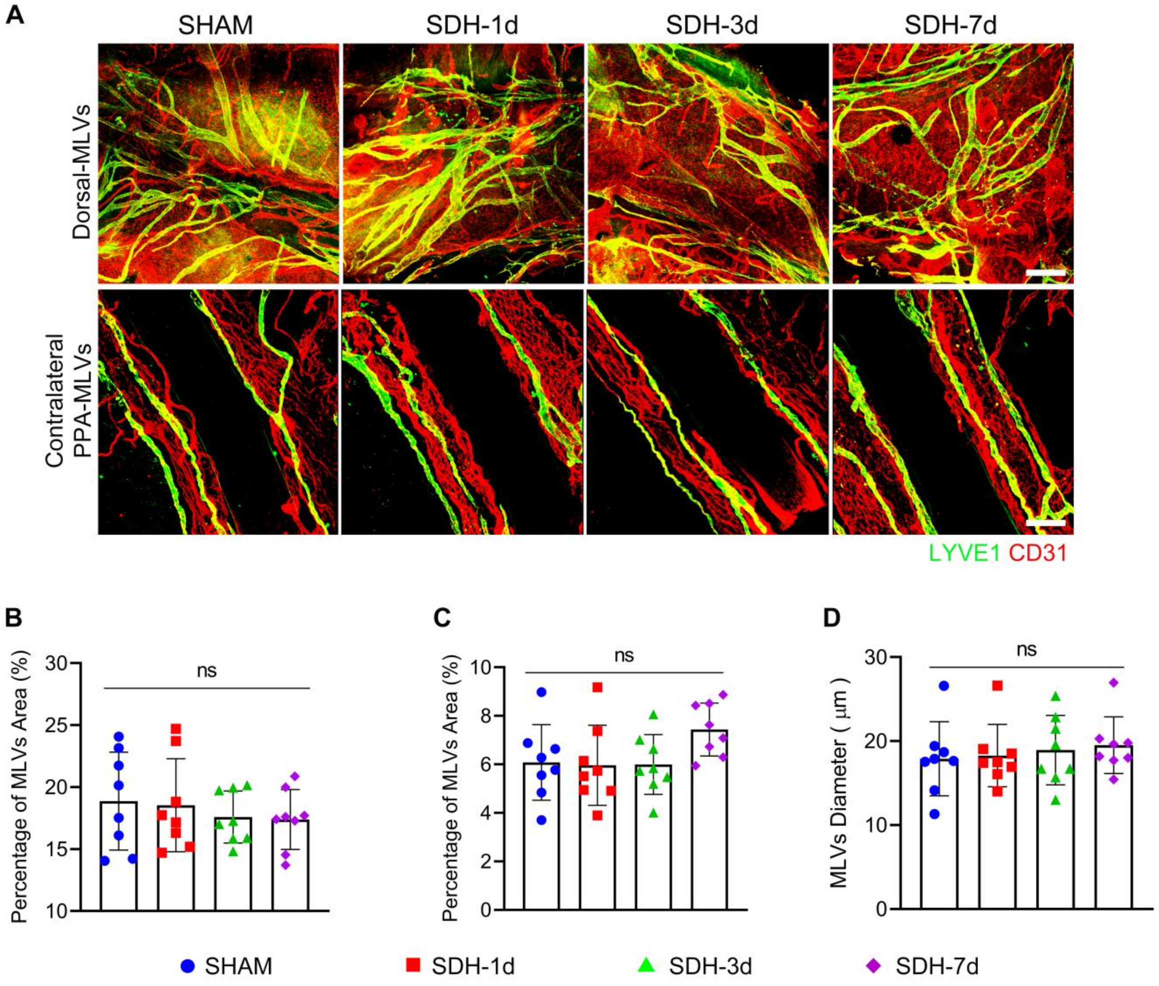

Figure S3. SDH does not cause evident pathological alterations in the dorsal and contralateral basal MLVs. (A) Immunostaining of the dorsal MLVs surrounding the TS and the contralateral basal MLVs adjacent to the PPA. **(B)** Quantifying the coverage area of the dorsal MLVs. n = 8 rats per group. Assessing the coverage area **(C)** and lumen diameter **(D)** of the contralateral basal MLVs. n = 8 rats per group. Data are presented as means ± SD, statistical analysis with one-way ANOVA followed by Dunnett post hoc test in **B-D**. ns, no significance. Scale bars: **A** 100μm.

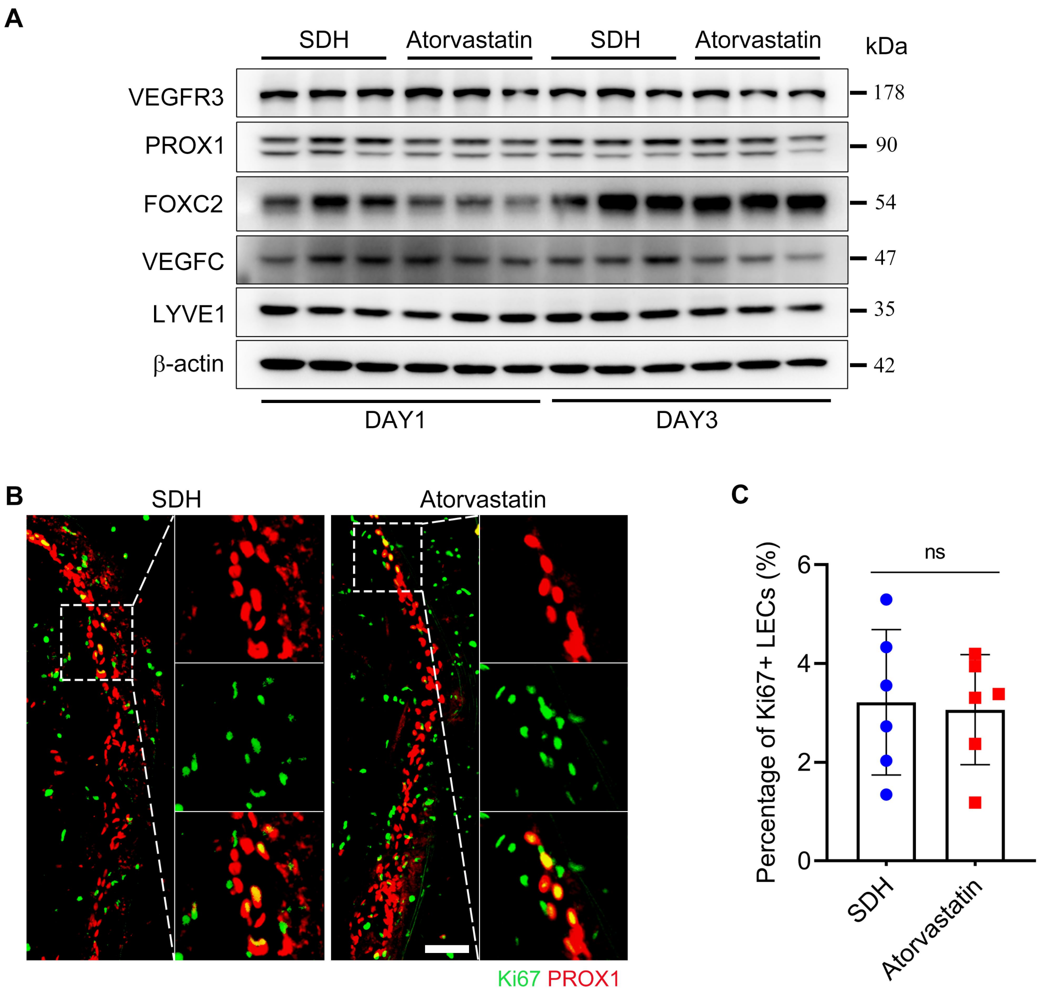

Figure S4. Atorvastatin treatment does not induce lymphangiogenesis in the meninges. (A) Western blot analysis of lymphatic marker protein expressions in the meninges following SDH with atorvastatin treatment. n = 3 rats per group. **(B-C)** Assessing meningeal lymphangiogenesis via Ki67+ LEC staining on the third day following atorvastatin treatment. n = 6 rats per group. Data are presented as means ± SD, statistical analysis with two-tailed unpaired Student's *t* test. ns, no significance. Scale bars: **B** 50μm.

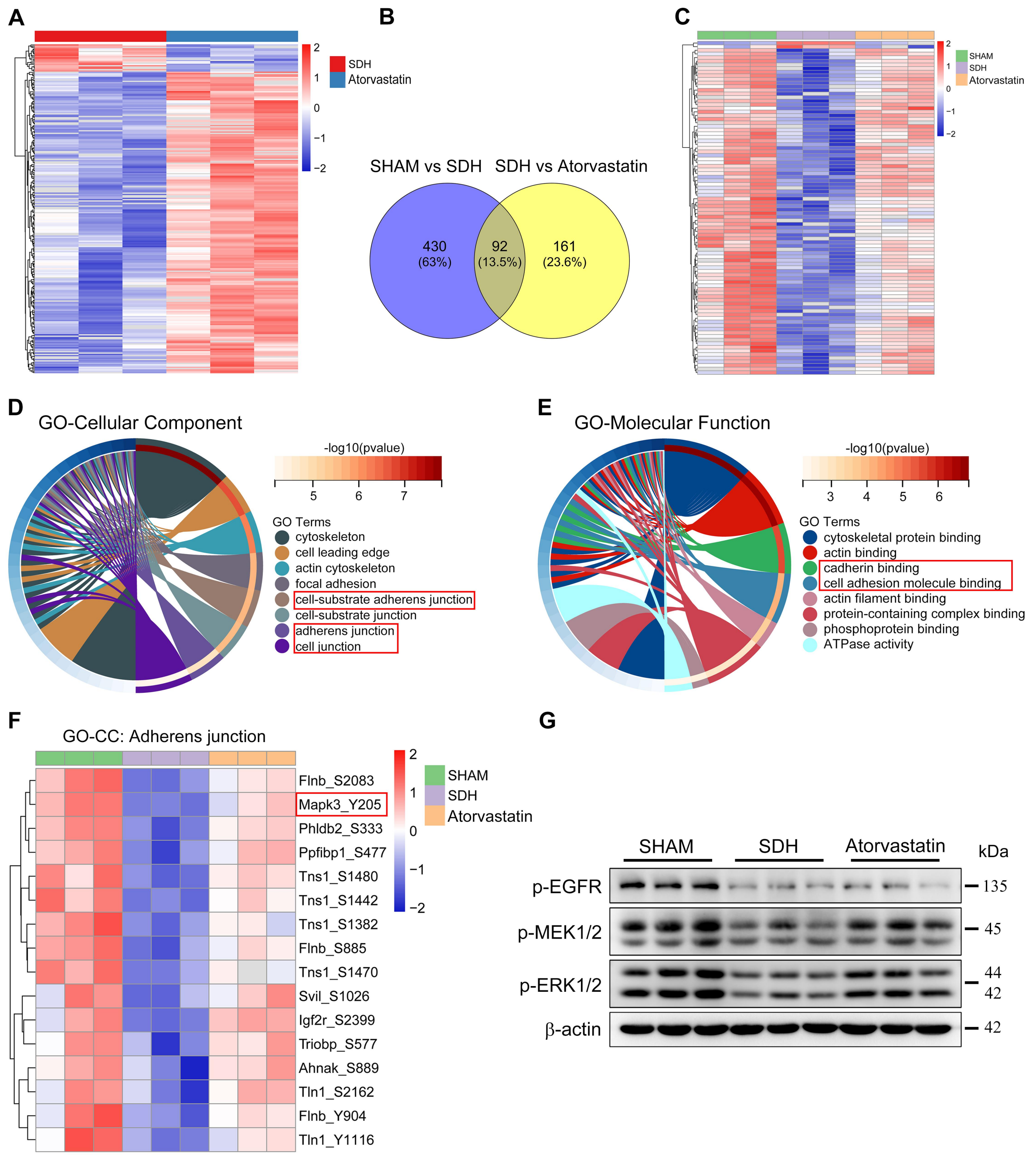

Figure S5. Atorvastatin treatment prevents the dephosphorylation of proteins in the meninges following SDH. (A) Heatmap showing relative phosphorylation levels of meningeal proteins in the atorvastatin treatment samples and SDH samples. **(B)** Venn diagram of overlapping proteins and their phosphorylation sites between the SHAM versus SDH group and SDH versus Atorvastatin group. **(C)** Heatmap exhibiting relative phosphorylation levels of these overlapped proteins in the three groups. The significant GO enrichment terms in cellular component **(D)** and molecular function **(E)** of these altered proteins in the atorvastatin treatment group compared to the SDH group. **(F)** Heatmap representation of the clustered proteins involved in adherens junctions and their specific phosphorylation sites. **(G)** Western blot analysis of the phosphorylation levels of EGFR, MEK1/2, and ERK1/2 in the meninges after atorvastatin treatment. n = 3 rats per group.

Table S1. List of antibodies used in this study

Antibody	Company	Catalog NO.	Host species	Dilution	Application
CD31	R&D	AF3628	Goat	1:200	IF, FC
LYVE1	Angiobio	11-036	Rabbit	1:100	IF
Podoplanin	Angiobio	11-035	Mouse	1:100	IF
FOXC2	Sigma-Aldrich	WH0002303 M2	Mouse	1:100; 1:1000	IF; WB
VE-cadherin	Sigma-Aldrich	#MABT886	Rabbit	1:100	IF
VE-cadherin	Santa Cruz	sc-9989	Mouse	1:200; 1:1000	IF; WB
α-catenin	ABclonal	A5635	Rabbit	1:500	WB
β-catenin	ABclonal	A19657	Rabbit	1:500	WB
δ-catenin	ABclonal	A11399	Rabbit	1:500	WB
VEGFR3	ABclonal	A13304	Rabbit	1:500	WB
VEGFC	ABclonal	A2556	Rabbit	1:500	WB
PROX1	ABclonal	A9047	Rabbit	1:500	WB
PROX1	Abcam	Ab199359	Rabbit	1:200	IF
LYVE1	Abcam	Ab183501	Rabbit	1:1000	WB
Ki67	Abcam	Ab279653	Mouse	1:200	IF
E-cadherin	CST	#14472	Mouse	1:1000	WB
ERK1/2	CST	#4695	Rabbit	1:1000	WB
phospho-ERK1/2 (Thr202/Tyr204)	CST	#4370	Rabbit	1:1000	WB,FC
phospho-MEK1/2 (Ser217/221)	CST	#9154	Rabbit	1:1000	WB
phospho-EGFR (Thr693)	Absin	abs130670	Rabbit	1:1000	WB
7AAD	Biolegend	420404	/	1:200	FC
CD45-PE/Cy7	Biolegend	202213	Rat	1:200	FC
PROX1- 488	Proteintech	CL488-67438	Mouse	1:100	FC
β-actin	ZSGB-BIO	TA-09	Mouse	1:2000	WB

Goat anti-Rabbit IgG-HRP	ZSGB-BIO	ZB-2301	Goat	1:5000	WB
Goat anti-Mouse IgG-HRP	ZSGB-BIO	ZB-2305	Goat	1:5000	WB
Donkey anti-Rabbit IgG Alexa Fluor 405	Abcam	Ab175649	Donkey	1:2000	FC
Donkey anti-Rabbit IgG Alexa Fluor 488	Invitrogen	A-21206	Donkey	1:1000	IF
Donkey anti-Rabbit IgG Alexa Fluor 555	Invitrogen	A-32794	Donkey	1:1000	IF
Donkey anti-Mouse IgG Alexa Fluor 488	Invitrogen	A-32766	Donkey	1:1000	IF
Donkey anti-Mouse IgG Alexa Fluor 594	Invitrogen	A-21203	Donkey	1:1000	IF
Donkey anti-Goat IgG Alexa Fluor 647	Invitrogen	A-32849	Donkey	1:1000	IF, FC
Donkey anti-Goat IgG Alexa Fluor 546	Invitrogen	A-11056	Donkey	1:1000	IF
DAPI	Invitrogen	D1306	/	1:10000	IF
